# 

**Published:** 1881-10

**Authors:** 


### WANTED,

Copies of the January number of this Journal: thirty cents each will be paid for the same. Gentlemen wishing to dispose of this number, will please forward to 2109 Chestnut Street, Philadelphia. Write your name on outside of wrapper.

### "GEMIASMA VERDANS."

Physicians wishing the 30th potency of the above remedy *to prove*, can have some by applying to Dr. Swan, No. 13 West Thirty-eighth Street, New York. This is one of the cryptogamia that cause chills and fevers; any person inhaling the odor of the ripe plant will surely have chills and fever.

VOL. I. OCTOBER, 1881. No. 10.

### THE HOMŒOPATHIC PHYSICIAN,

A MONTHLY JOURNAL OF MEDICAL SCIENCE.

*"Magna est veritas, et prævalebit."*

E. J. LEE, M. D.
EDITOR.

[illegible]

CONTENTS:
(See inside of Cover.)

CONTRIBUTORS:

Drs. H. C. Allen, T. F. Allen, Edward Bayard, J. B. Bell, E. W. Berridge, Geo. H. Clark, A. C. Cowperthwaite, Ad. Fellger, Wm. J. Guernsey, W. A. Hawley, John Hall, W. M. James, W. H. Jenny, Ad. Lippe, C. Lippe, Malcolm MacFarlan, Thos. Moore, C. F. Nichols, C. Pearson, T. F. Pomeroy, Leila A. Rendell, C. Carleton Smith, P. P. Wells, Wm. P. Wesselhoeft, David Wilson, (London), T. P. Wilson, and many others.

NOTICE.

***All articles for publication, books for review, business communications, etc., must be sent to Editor, 2109 Chestnut Street, Philadelphia.***

PHILADELPHIA:

N. B.—Authors of accepted papers are furnished with copies of the number in which their articles appear; application for such copies to be made when sending papers. Any irregularity in the receipt of this Journal should be promptly reported to Editor.

A prize of fifty dollars will be awarded to author of the best essay printed in this Journal for the year 1881. To be adjudged by three physicians of note.

